# The effect of art therapy on quality of life and fatigue in breast cancer patients: a meta-analysis

**DOI:** 10.3389/fonc.2025.1495887

**Published:** 2025-05-01

**Authors:** Yujing Zou, Shanshan Zhai, Jiawen Dong, Tianyu Zhao, Jin Yang

**Affiliations:** School of Clinical Medicine, Hangzhou Normal University, Hangzhou, China

**Keywords:** mental health, alternative therapy, psychotherapy, cancer, meta-analysis

## Abstract

Breast cancer imposes a substantial global health burden, with survivors frequently experiencing post-treatment quality of life deficits and chronic fatigue. While art therapy has gained recognition as a potential adjuvant intervention, its therapeutic value remains debated. To evaluate art therapy’s efficacy, we conducted an exhaustive search of major electronic databases through October 2023, identifying 22 eligible RCTs reported in 22 papers that met predefined inclusion criteria from major databases including Web of Science Core Collection, Cochare, Embase, and Pubmed. Study quality was assessed using Cochrane risk-of-bias tools, with data extraction and analysis performed in RevMan 5.3. In this meta-analysis, we aimed to investigate the effect of art therapy on quality of life and fatigue in breast cancer patients. As of October 2023, relevant randomized controlled studies were searched from relevant electronic databases and articles were screened, quality evaluated, and data related to quality of life and fatigue were extracted and analysed using RevMan 5. 3. A total of 22 randomized controlled studies from 22 papers were included in this analysis. The results obtained according to this analysis included the effect of art on quality of life in breast cancer: SMD=0.38 (95% CI, 0.25 to 0.51; *P*≤0.00001), the fatigue-related indices: SMD=-0.03 (95% CI, -0.21 to 0.15; *P*=0.73). The effect of intervention time less than two months: SMD=0.21 (95% CI, -0.01 to 0.43; *P*=0.07), more than or equal to two months: SMD=0.45 (95% CI, 0.28 to 0.62; *P*<0.00001). Different types of interventions: art therapy in painting and calligraphy: SMD=0.09 (95% CI, -0.17 to 0.35; *P*=0.49), music-included art therapy: SMD=0.51 (95% CI, 0.32 to 0.70, *P*<0.00001), music-out-of-treatment art therapy: SMD=0.44 (95% CI, 0.19 to 0.69; *P*=0.0005). Follow-up time greater than or equal to six months: SMD=0.14 (95% CI, -0.09 to 0.36; *P*=0.22), follow-up time less than six months: SMD=-0.34 (95% CI, -0.64 to -0.04; *P*=0.03). In summary, this analysis demonstrated that art therapy improved quality of life, but the effects of fatigue reduction only occur within six months, with the degree of the benefits being correlated with the type, length, and timing of the intervention.

**Systematic review registration:**

https://www.crd.york.ac.uk/PROSPERO/myprospero, identifier CRD42023476055.

## 1 Introduction

Breast cancer constitutes the leading malignancy and primary oncologic mortality cause in women globally (1). WHO surveillance data position it as the most prevalent neoplasm worldwide, surpassing lung cancer incidence rates, thus establishing breast cancer management as a critical global health challenge requiring urgent multidisciplinary attention. Conventional surgical resection, adjuvant chemotherapy, radiation therapy, and other therapies are used to treat breast cancer. But these surgical techniques frequently result in adverse consequences including anxiety, sleeplessness, and exhaustion. Relevant research has revealed that breast cancer patients have a much worse quality of life than the general population, which will have a major effect on the patients’ day-to-day lives (2). There are two main causes of the side effects: first, the surgery’s aftermath damages the patient’s self-esteem and perception, which causes sleeplessness, anxiety, and other dysfunctions (3). Secondly, the patient’s own fear of cancer and uncertainty about the outcome of the treatment may exacerbate a number of psychological disorders, including depression, fatigue, and other psychological disorders. Thus, in order to enhance and restore patients’ feeling of well-being, it is critical to concentrate on the side effects of breast cancer and look for beneficial and efficient therapies to improve tiredness, sleep, and quality of life.

Art therapy has been defined as “a form of psychotherapy in which art media are used as the primary means of expression and communication with the goal of enabling the recipient to change and grow on a personal level through the use of art materials in a safe and accessible environment” by the British Association of Art Therapists (4), and its non-invasive, secure, and adaptable qualities have drawn interest in recent years. Painting, music, dance, and other media are examples of art therapies. It is noteworthy that in this study, yoga was also included in this category because yoga encourages self-expression of the individual through the integration of the body and mind through forms of asana, breathing, and meditation, which is in line with the definition and goals of art therapy.

A growing body of systematic reviews has established consensus regarding the therapeutic potential of art-based interventions in breast cancer care, with particular emphasis on their benefits for health-related quality of life (QOL) (5, 6). Notably, a randomized controlled trial highlighted tango therapy’s unique benefits for dance-naïve patients, showing significant improvements in cancer-related fatigue (CRF) scores and QOL indices through structured dance protocols (7).However, the academic discourse reveals notable discrepancies in empirical findings across different art therapy subdomains. Contrary to movement-based approaches, a clinical trial evaluating creative art therapy demonstrated no statistically significant reduction in generalized fatigue among breast cancer survivors at one-month post-radiation follow-up (8). This therapeutic incongruity extends to mind-body interventions, where a study examining yoga-integrated art therapy failed to replicate the fatigue-alleviating effects observed in other somatic modalities (9).

Given the substantial heterogeneity in existing evidence and the limited evidence confirming mood-related outcomes of specific art therapy modalities, a focused meta-analysis was conducted to clarify art therapy’s precise effects on quality of life and fatigue in breast cancer populations. To address evidence gaps, this analysis introduces a novel dimensional framework by implementing subgroup stratification. First, art interventions were categorized into process-oriented modalities (self-expressive painting/writing) versus receptive-based modalities (music/yoga engagement) to elucidate mechanistic differences in therapeutic action. Second, multi-temporal assessments (≤2 or >6 months post-intervention) were incorporated to map the trajectory of therapeutic benefits.

## 2 Method

We registered on the PROSPERO website in October 2023, and provided the registration number CRD42023490915.

### 2.1 Literature and research

The database used for this study is derived from major electronic databases, and we consulted electronic databases such as the Web of Science Core Collection, Cochare, Embase, Pubmed, and other databases. To construct a reasonable formula for searching for relevant research literature while searching literature, it is possible to use ‘xxx’ to search for relevant research literature, like Pubmed(“art therapy” [MeSH Terms] OR “Art Therapies” OR “Art Therapies” OR “Music” [MeSH Terms] OR “Music Therapy” OR “Therapy, Music” OR “paint” [MeSH Terms] OR “paint” OR “drawing” OR “drawings”OR “draw”OR “Dance” [MeSH Terms] OR “Dance Therapy” OR “Dance Therapies” OR “Drama Therapy” [MeSH Terms] OR “Dramatherapy” OR “Supportive-Expressive therapy” OR “Writing therapy”) AND (“Breast Cancer” [MeSH Terms] OR “Mammary Cancer” OR “Breast Neoplasms” OR “Breast Tumor” OR “Cancer of the Breast”) AND((“Quality of Life” [MeSH Terms] OR “Life Quality” OR “HRQOL” OR “Quailty of sleep”) OR (“fatigue” [MeSH Terms] OR “Lassitude”)).

### 2.2 Inclusion and exclusion criteria

For the subsequent screening of qualified literature, all the initial obtained literature was imported into EndNote X9. The researchers devised inclusion and exclusion criteria that were consistent with the PICOS principle during literature screening. The inclusion criteria are: (1) the experiment type must be randomized controlled (RCT); (2) the experimental subjects are women who have breast cancer; (3) the intervention must be art intervention without limiting the type of art used, including music, painting, yoga and so on, and the control measure is conventional treatment without art intervention; (4) the full text of the literature can be obtained, and the mean and standard deviation of the experimental group and the control group can be provided or calculated; (5) the indicators used to measure the outcomes included PSQI, ISI, MOS-sleep, WHO, FACT-G, FACT-B, QoL, QLQ-C30, and others. The exclusion criteria were: (1) exclusion of non-RCT trials, reviews, systematic evaluations, Meta-analyses, and animal experiments; (2) exclusion of literature that did not meet the literature inclusion criteria. Two researchers conducted an independent double-blind screening of the qualified literature, following the criteria, and a third researcher was hired to make a decision on controversial articles.

### 2.3 Quality evaluation

**Figure 1:**
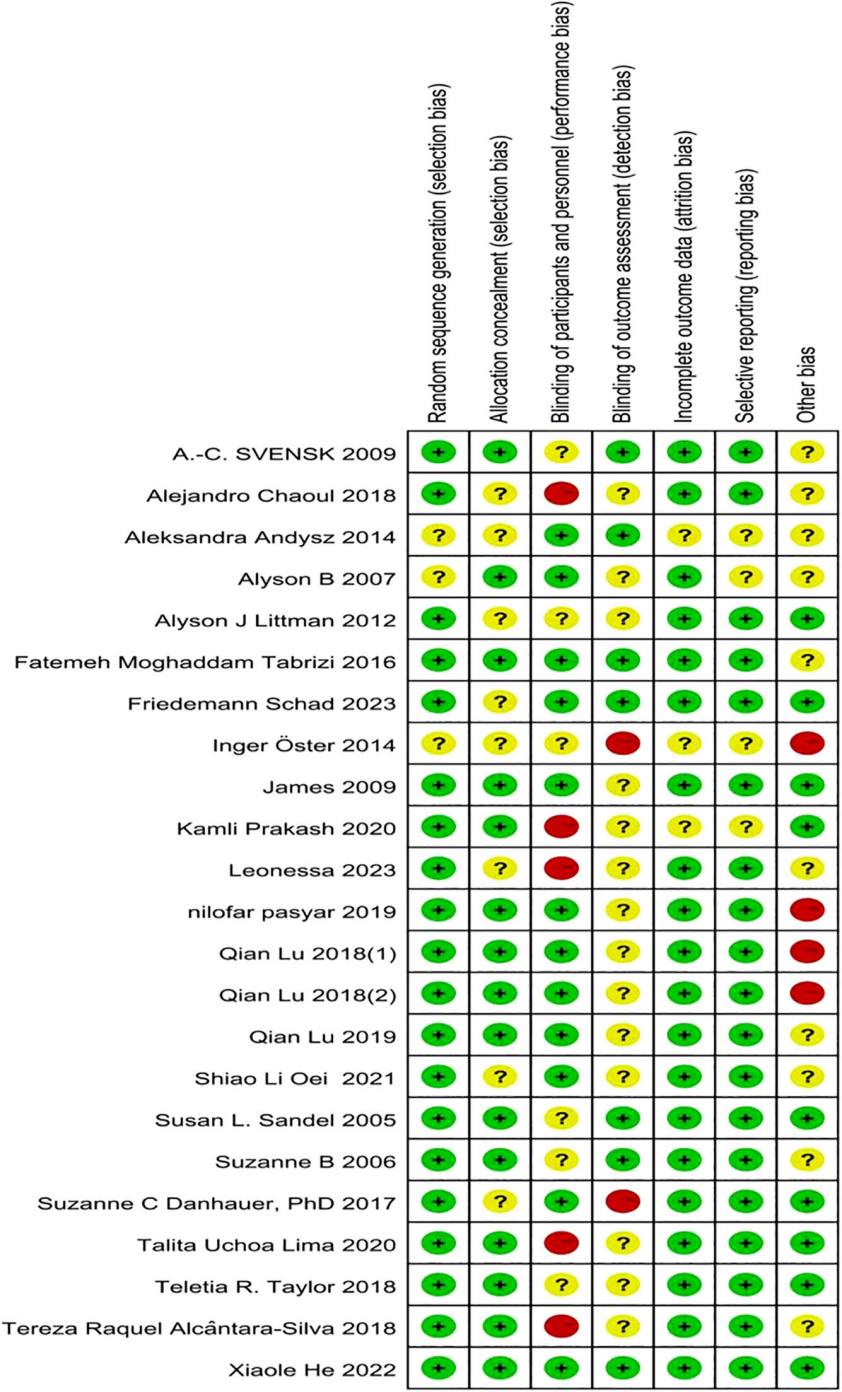
The risk-of-bias assessment for each of the included studies.

The study’s literature was evaluated from seven aspects using Review Manage 5. 3 software’s Cochrane system evaluation ‘risk bias evaluation’ tool:fixed allocation method, hidden allocation method, blind subjects, blind result evaluator, data result integrity, selective reports and other biases,and each quality evaluation item was evaluated according to three categories: low risk, unclear and high risk. 22 included studies were assessed for risk of bias based on the criteria (**Figure 1**). In random sequence generation, 19 studies were low risk with guaranteed grouping randomization; 3 were uncertain with further confirmation of methodological reliability; in allocation concealment, 15 were low risk with effective prevention of information leakage; 8 were uncertain with unclear situation; in participant and researcher blinding, 12 were low risk with blinding implementation in place; 6 were uncertain with ambiguous blinding situation; and 5 were high risk with subjective factors Highly influenced. For outcome assessment blinding, 7 low risk, good test objectivity; 14 uncertain; 2 high risk, susceptibility to bias in test results; for incomplete outcome data, 19 low risk, complete data; 3 uncertain; for selective reporting, 18 low risk, standardized reporting; 4 uncertain; and for other bias, 9 low risk, good overall; 10 uncertain; 4 high risk, potential bias potential.

### 2.4 Statistical methods

Data was arranged with assistance from Review Manager (RevMan) version 5.3. Different scales were used in the studies to measure quality of life and fatigue. Indicating that these studies should be standardized before being included in the meta-analysis, the effect size of the standardized mean difference was expressed as Cohen’s d [95% confidence interval]; therefore, continuous variables were reported as scale scores and standardized mean difference (SMD). The Cochrane Collaboration was utilized to conduct meta-analyses of key fixed-effect outcomes, which revealed heterogeneity and expressed it in terms of I^2^ and effect sizes. I^2^ indicates the proportion of the total variation that is reflecting differences due to non-sampling errors. with I^2^ less than 25%, 50%, and 75% indicating low, medium, and high heterogeneity, respectively, so the choice of model for the analysis of the data therefore depended on the magnitude of I^2^. If I^2^ exceeds 50%, it means there is significant heterogeneity and a random effects model is employed. If I^2^ ≤ 50%, a fixed effects model is used. Small, medium, and large effect sizes were interpreted as Cohen’s d < 0.2, 0.2 ≤ d < 0.5, and d > 0.5. Statistical significance (p-value) was set at 0.05, and the combined results are presented graphically as forest samples.

## 3 Results

### 3.1 Assessment of identified trials

**Figure 2:**
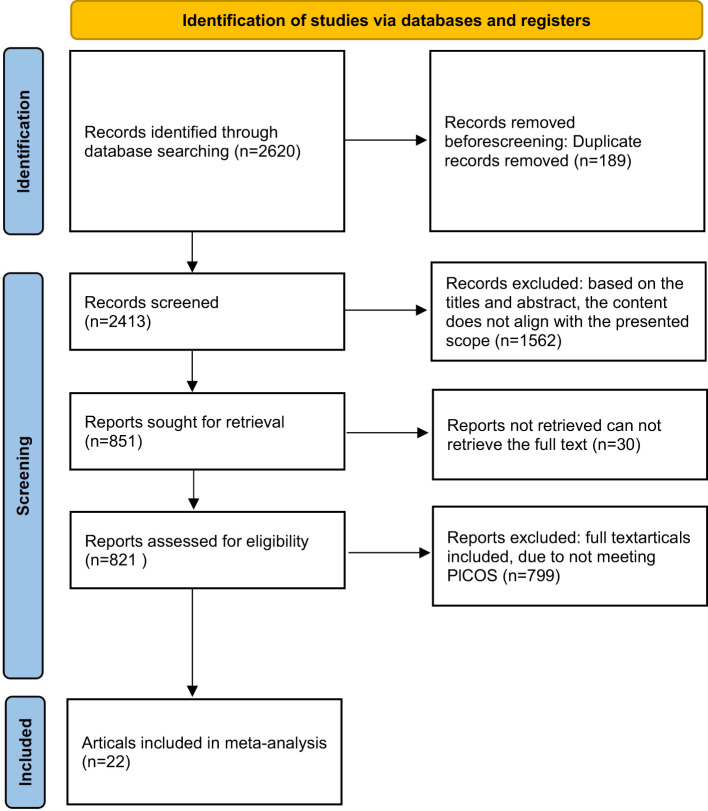
Literature screening flowchart.

As shown in **Figure 2**, which is a flow chart of literature screening based on the PRISMA principle.we found a total of 2,620 articles in the database and eliminated 189 duplicates. 1562 articles were excluded based on their titles and abstracts. There are 851 remaining articles, 30 of which can’t retrieve the full text. Eligibility was assessed for 821 articles, and 799 were excluded due to exclusion criteria, resulting in a quantitative synthesis of 22 documents. Nine of the literature were related to fatigue and 15 were related to quality of life (**Figure 2**).

### 3.2 Characterization of the literature

In total, there were 9 studies from the United States, 2 from Iran, 2 from Beazil, 3 from China, 1 from the United Kingdom, 2 from Germany, 1 from India, and 2 from Australia. All of the study types were RCTs. The studies included only female breast cancer patients. **Table 1** describes the details of the interventions, which included 14 dynamic art therapies and 8 static art therapies. The former included yoga, dancing, or tango, and the latter included writing with positive psychological cues, supportive expression, drawing, and listening to music. All the art therapy interventions were under the supervision of qualified therapists. The length of the interventions were all focused on two to six months, with the two studies in LU having a shorter intervention of three weeks. The measure for the control group was usual care, but the measure for the control group in both of lu’s studies was write about their cancer facts unrelatedtoemotions, which we believe was set up to interfere with the factor of writing for the control. The primary outcome indicators were quality of life and fatigue index. The former indicator article measured with the QLQ-C30, FACT-B, FACT-G, QOL and WHOQOL-BREF scales, and the latter indicator was expressed with the FACT-F, BFI scale. The specific ones are shown in **Table 1**.

**Table 1:** Details of included studies.

Reference	Study design	Intervention type, time	Control	Measure time	Main outcome	Tools	Site
**Alejandro Chaoul 2018 (** 10)	RCT	Tibetan Yoga	usual care	Baseline 1-week 3-mounth 6-mounth 12-mounth	Fatigues	BFI	America
**Alyson J Littman 2012 (** 11)	RCT	Yoga, 6 months	Wait list controls	Baseline, 6month	Fatigue, quality of life	FACT-F and FACT-G	America
**Fatemeh Moghaddam Tabrizi2016 (** 12)	RCT	Supportive expressive discussion groups, 12weeks	Routine care	Baseline, 8-week	Fatigue	EORTCQLQ-C30	Lran
**James2009 (** 13)	RCT	Yoga, 3 months	Usual care	Baseline 3-Month	Fatigue	0-9 scales	America
**Leonessa 2023 (** 14)	RCT	Yoga and Belly Dance, 16weeks	Education and continue usual care	16-week, 6-month, and 12-month	Fatigue	FACT-F	Australia
**Nilofar Pasyar2019 (** 15)	RCT	Yoga, 8weeks	Standard routine care	baseline, 4th and 8th week.	Life quality	EORTC QLQ_C30,	Iran
**Shiao Li Oei1, 2021 (** 16)	RCT	Tango, 6weeks	Wait list controls	6-month,12-month	Fatigue, life quality	EORCT - QLQ 30, CFS-D,	Germany
**Suzanne C Danhauer, PhD2017 (** 17)	RCT	Yoga, 10weeks	Education.	baseline, 5-week, 10-week	Fatigue, life quality	FACT-F,FACT–B	America
**Teletia R. Taylor 2018 (** 18)	RCT	Yoga, 8weeks	Wait list controls	Baseline, 8-week	Fatigue	BFI	America
**A.-C. SVENSK 2009 (** 19)	RCT	Painting, 5weeks	Wait list	Baseline, 2-month, 6-month	Life of quality	WHOQOL-BREF	America
**Friedemann Schad 2023 (** 7)	RCT	Tango program 6 weeks	Routine activities	baseline and 6-week	Self-reported fatigue and further quality of life	EORTC-QLQ-C30	Germany
**Inger Öster 2014 (** 20)	RCT	individualised art therapy sessions 5 weeks	Routine care	5/7-year	Quality of life	WHOQOL-BREF	Australia
**Kamli Prakash 2020 (** 21)	RCT	yoga 4 months	Routine care	1,2,3,4,5,and 6th cycles of chemotherapy	Quality of Life	QLQ-C30	India
**LU QIAN 2019 (** 22)	RCT	PTC 4 weeks	CFC	1.month 2.month	Quality of Life	FACT-B	China
**Qian Lu 2018 (** 23)	RCT	SRC,ESRC 3 weeks	CFC	baseline, 1.month 2.month 3.month 4.6-month	Quality of life	FACT-G	China
**Susan L. Sandel 2005 (** 11)	RCT	Movement and Dance 12 weeks	wait list controls	13.week 26-week	Quality of Life	FACT-B	America
**Suzanne B 2006 (** 24)	RCT	music 15 weeks	usual oncology and supportive care	baseline 5.week 3-month	Quality of Life	FACT-G	England
**Talita Uchoa Lima 2020 (** 25)	RCT	Music 9 weeks	usual care	each session of the first 3 CT cycles	Quality of life	WHOQOL-BREF	Brazil
**Tereza Raquel Alcântara-Silva 2018 (** 26)	RCT	Music 5 weeks	Wait-list	the first week of radiotherary, on the week of intermediary, last week of radiotherapy(5-week)	Fatigue,quality of life	FACT-F,FACT-G,	Brazil
**Xiaole He 2022 (** 27)	RCT	Dance programme 16 weeks	general health consultant	17-week	Fatigue	BFI	China
**Aleksandra Andysz 2014 (** 28)	RCT	Yoga 10 weeks	Wait-list controls	10-week	Quailty of life	QLQ-C30 and QLQ-BR23	America
**Alyson B 2007 (** 29)	RCT	Yoga 12 weeks	Wait list control	baseline, 1-month 3-months	Quailty of life	FACT-General	America

CFC, a cancer-fact condition to write about facts relevant to the cancer experience; PTC, a benefit-finding condition to write about positive aspects of their cancer experience; SRC, a self-regulation condition to write about deepest feelings; ESRC, enhanced self-regulation condition; BFI, brief fatigue inventory; FACT-F, Functional Assessment of Cancer Therapy—Fatigue; FACT-G, Functional Assessment of Cancer Therapy—general; CFS, cancer fatigue scale; FACT-B, Functional Assessment of Cancer Therapy–Breast; BFI, Brief Fatigue Inventory; WHOQOL-BREF, World Health Organization Quality of Life-BREF; EORTCQLQ-C30, The European Organization for Research and Treatment of Cancer QoL Core Questionnaire.

### 3.3 Primary outcome

**Figure 3:**
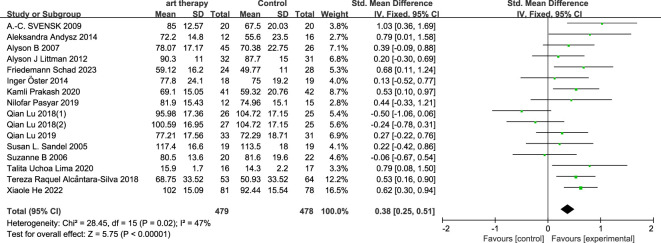
Influence of art therapy on quality of life of breast cancer patients.

**Figure 4:**
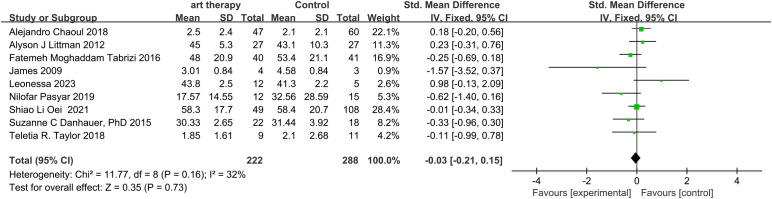
Influence of art therapy on fatigue of breast cancer patients.

The total SMD of the effect of art on quality of life in breast cancer was 0.38 (95% CI, 0.25 to 0.51; *P*≤0.00001) (**Figure 3**). The total SMD of the effect of art therapy on fatigue-related indices in breast cancer patients was -0.03 (95% CI, -0.21 to 0.15; *P*=0.73) (**Figure 4**). In sensitivity analyses, excluding any of the articles, the two final results of the outcome indicators did not change significantly. The quality of life-related indicators experienced a decrease in heterogeneity from 47% to 24% as a result of the exclusion of one article (23). The heterogeneity dropped from 32% to 18% when one article study related to fatigue (14) was excluded.

#### 3.3.1 Subgroup analysis on quality of life

**Figure 5:**
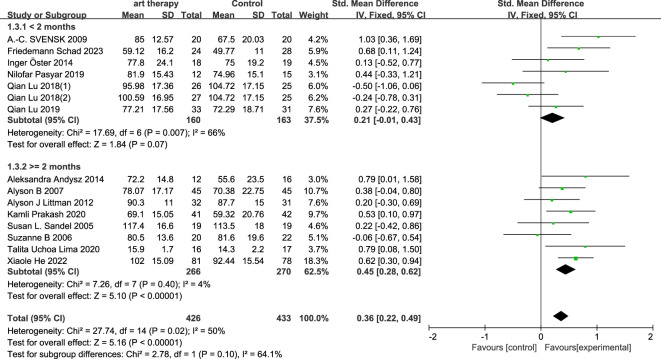
According to the types of intervention, the quality of life was analyzed in subgroups.

**Figure 6:**
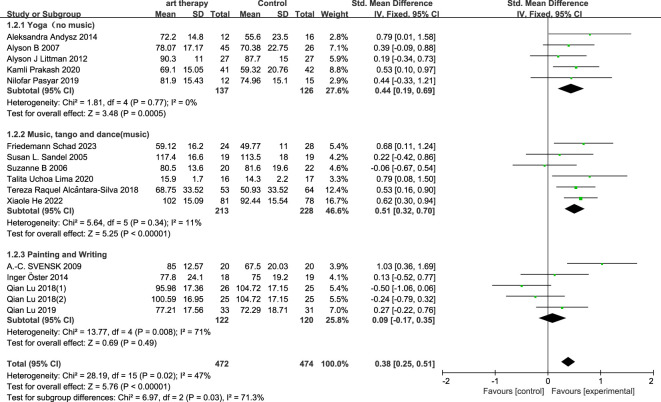
According to the intervention duration, the quality of life was analyzed in subgroups.

In the subgroup analysis of intervention length, art therapy with an intervention length of less than two months had an SMD of 0.21 (95% CI, -0.01 to 0.43; *P*=0.07) on quality of life for breast cancer patients, and with an intervention length of more than or equal to two months it had an overall SMD of 0.45 (95% CI, 0.28 to 0.62; *P*<0.00001) on breast cancer (**Figure 5**). In the subgroup categorization of art therapy domains, the total SMD for art therapy in the painting and writing category was 0.09 (95% CI, -0.17 to 0.35; *P*=0.49), the SMD for art therapy that included music (music, tango, and dancing) was 0.51 (95% CI, 0.32 to 0.70, *P*<0.00001), and art therapy that did not include music (yoga) had a SMD of 0.44 (95% CI, 0.19 to 0.69; *P*=0.0005) (**Figure 6**).

#### 3.3.2 Subgroup analysis on fatigue

**Figure 7:**
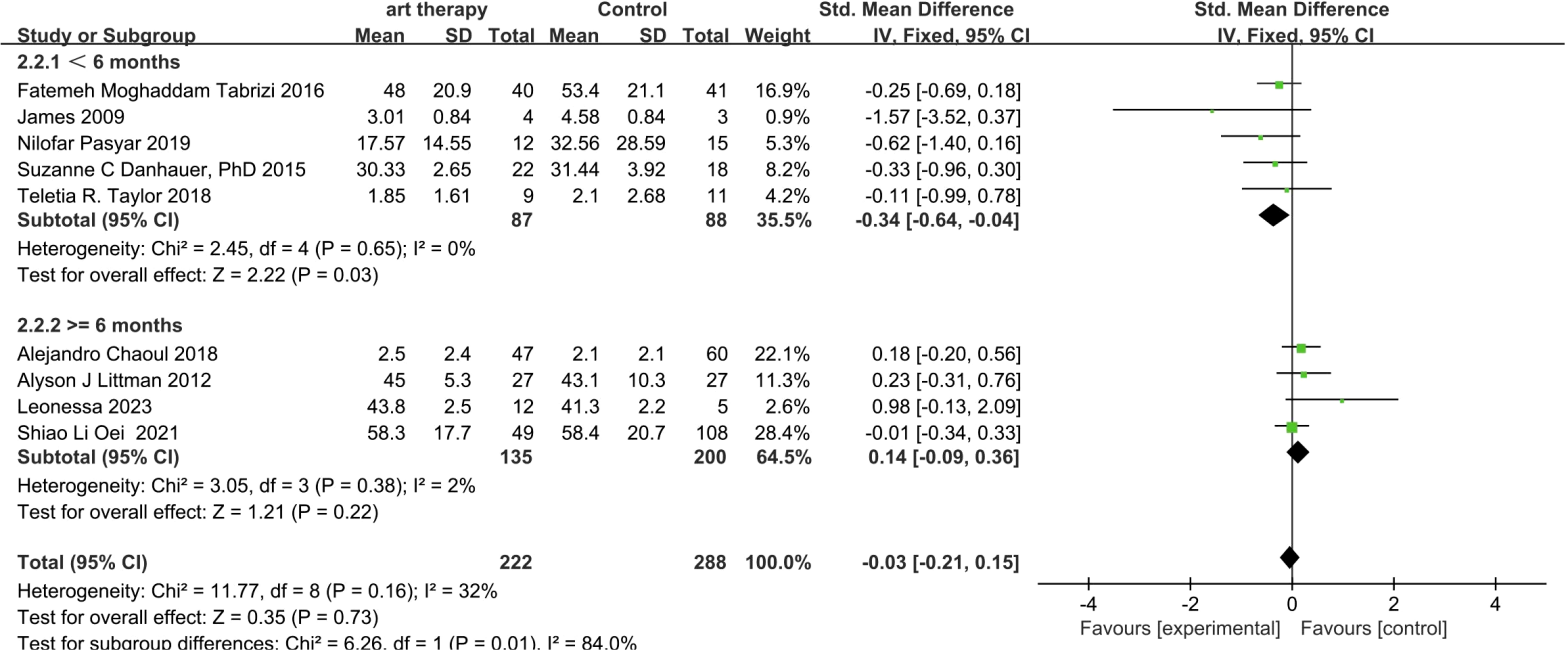
According to the follow-up time, fatigue was analyzed in subgroups.

In the subgroup analysis of follow-up time, the SMD was -0.34 (95% CI, -0.64 to -0.04; *P*=0.03) for a follow-up length of less than 6 months and 0.14 (95% CI, -0.09 to 0.36; *P*=0.22) for a follow-up length of more than 6 months (**Figure 7**).

### 3.4 Heterogeneity test

In this analysis it is known that the duration of follow-up is a source of heterogeneity in the effect of art therapy on fatigue-related indices in breast cancer. The length of intervention and the type of art were sources of heterogeneity in the effect of art therapy on the quality of life aspects of breast cancer.

### 3.5 Quality evaluation

Using Cochrane system evaluation “risk bias assessment” tool to evaluate the quality of 22 articles included in the study, it is found that the vast majority of articles use random control method to test, and most of the hidden assignments are low-risk, and others are not clearly described in the articles (10, 20), and the blind method for participants is half low-risk and unclear (11, 18–20, 24, 30), and account for the same proportion (14, 25, 26), while the blind low-risk (7, 11, 12, 19, 24, 27, 28), and high-risk (17, 20) for the outcome evaluator are less, and the rest are not mentioned in the articles on the integrity of the outcome data (20, 21, 28, 29). The overall quality of the included documents is optimistic, which may have little impact on the reliability of the results (**Figure 1**).

### 3.6 Publication bias

**Figure 8:**
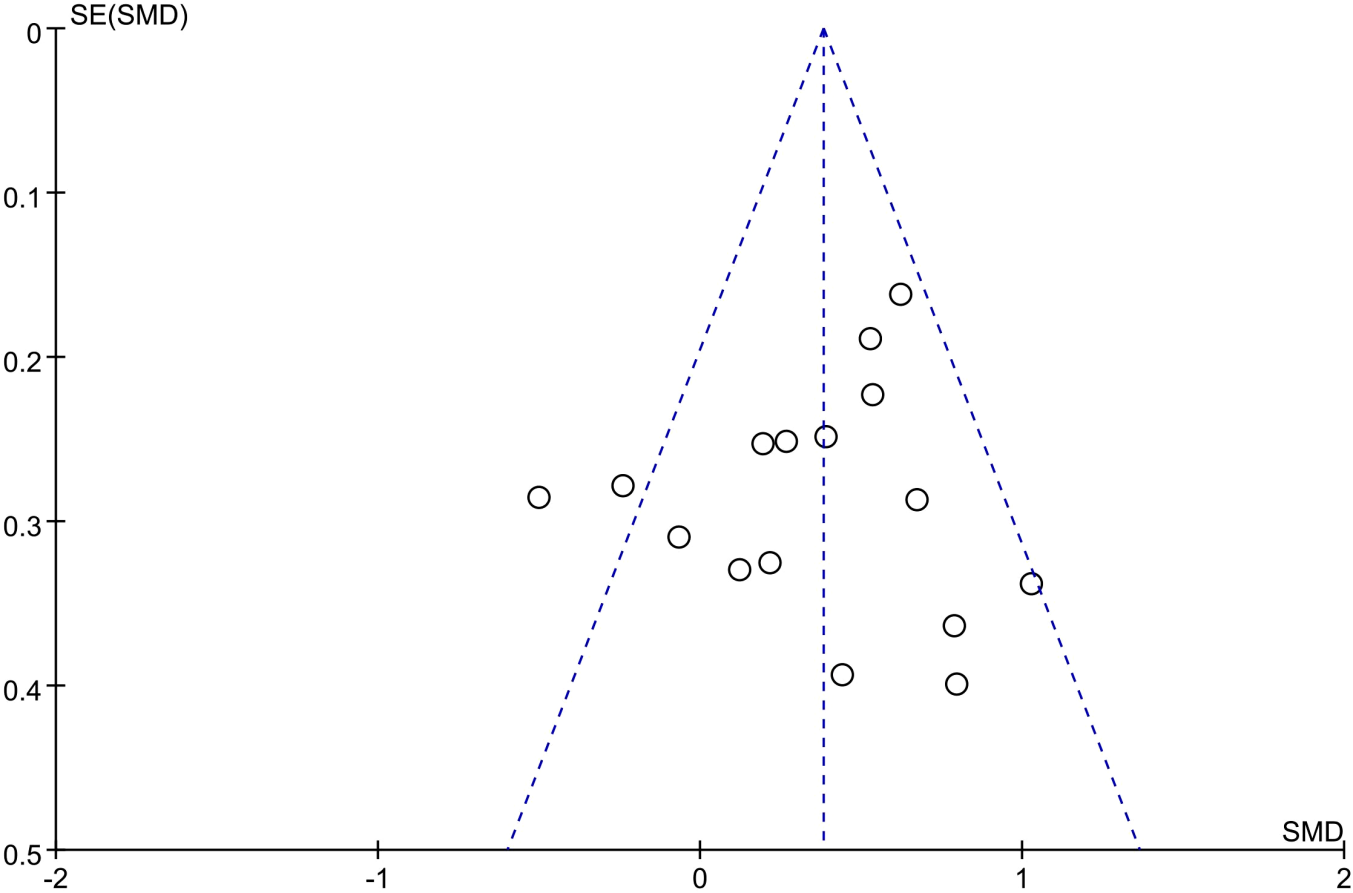
Publication bias funnel plot of the effect of art therapy on the quality of life of breast cancer patients.

**Figure 9:**
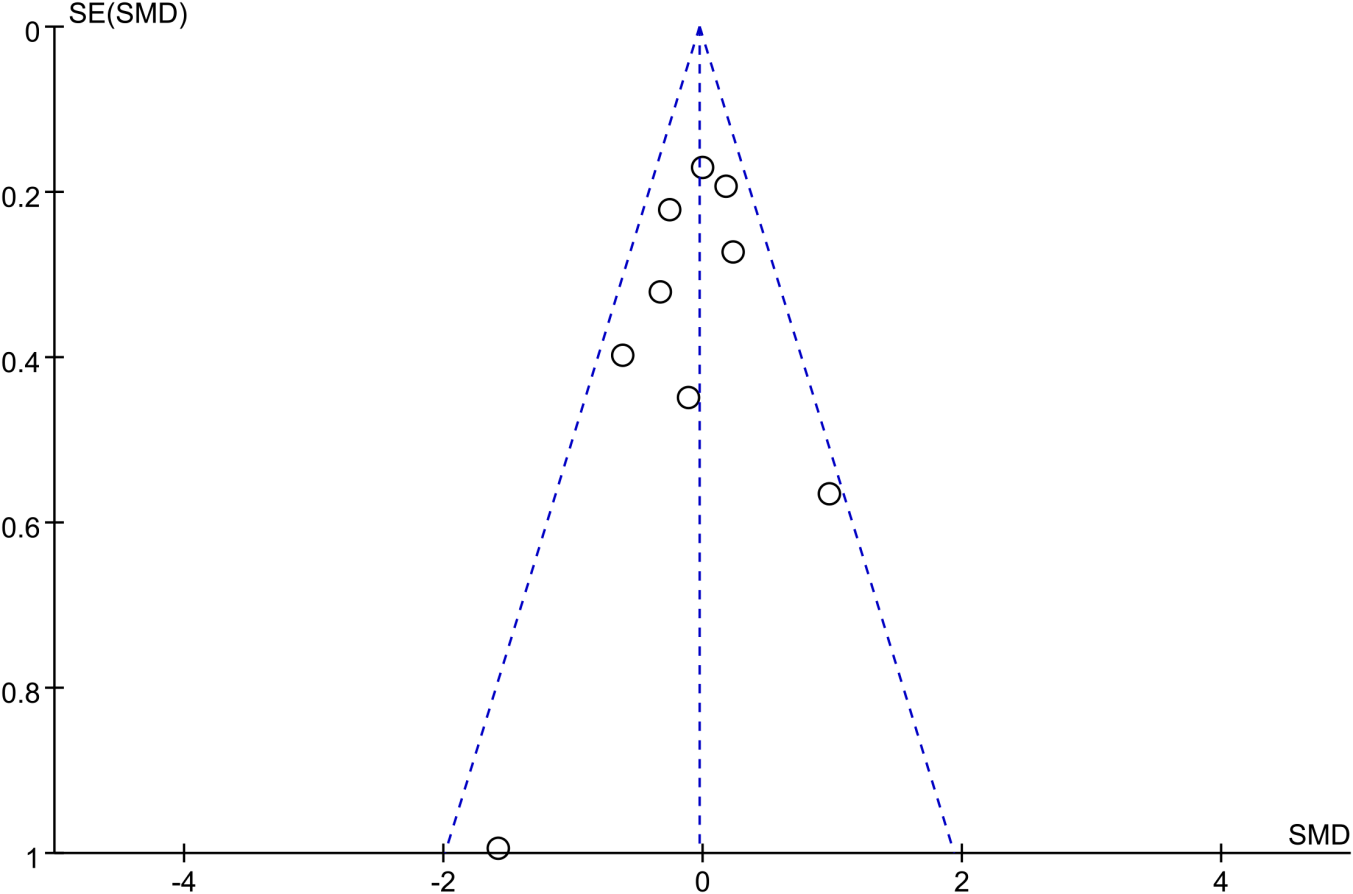
Publication bias funnel plot for the effect of art therapy on fatigue in breast cancer patients.

The main outcome indicators (quality of life, fatigue) of this study were analysed separately using the bias funnel plot approach for the scores of breast cancer patients, and the results showed that the scatters were uniformly distributed on both sides and the graphs were symmetrical, indicating that there is a low possibility of publication bias in this study (**Figures 8**, **9**).

## 4 Discussion

A prior meta-analysis supported the findings of the current meta-analysis, which indicated that art therapy significantly improved quality of life but had no effect on fatigue (31). For quality of life, duration-stratified analysis showed interventions ≥2 months achieved clinically meaningful improvements (SMD=0.45, 95% CI 0.28-0.62, *P*<0.00001) compared to shorter interventions (SMD=0.21, 95% CI -0.01-0.43, *P*=0.07). Modality-specific effects emerged distinctly: music-based or movement-integrated modalities demonstrated superior efficacy (SMD=0.51, 95% CI 0.32-0.70; SMD=0.44, 95% CI 0.19-0.69) over static modalities (SMD=0.09, 95% CI -0.17-0.35, *P*=0.49). Fatigue outcomes exhibited time-dependent attenuation, with short-term follow-up (<6 months: SMD=-0.34, 95% CI -0.64–0.04, *P*=0.03) losing significance during extended follow-up (≥6 months: SMD=0.14, 95% CI -0.09-0.36, *P*=0.66). Temporal dynamics analysis suggests insufficient intervention duration (<2 months) may disrupt neurobiological adaptation processes, while extended follow-up (≥6 months) reveals therapeutic effect decay, highlighting the need for maintenance protocols.

We investigated the mechanisms underlying the results by reviewing the pertinent literature in light of the aforementioned findings. Mechanistically, our findings align with neuroendocrine evidence demonstrating art therapy’s capacity to modulate stress biomarkers. The observed cortisol reduction reflects HPA axis normalization, a critical pathway given the axis’ central role in stress regulation (32). Another study has shown that the neural effects of visual art production on psychological resilience in adulthood. Psychological resilience is conceptualized as a protective personality trait that enables individuals to control the negative effects of stress and thus function successfully and healthily even under stressful life conditions (33). Intervention efficacy variations may stem from neurobiological specificity: Rhythmic auditory stimuli (e.g., music therapy) activate striatal dopaminergic pathways (34). Similar to this, the beat of music can also have a “cellular massage effect” on tissue cells, causing the cells to resonate with the beat and create a harmonic sense that can enhance the performance of several systems and enhance patients’ quality of life. The lack of effectiveness of the art therapy intervention of drawing and writing alone may be due to the inability to control subjectivity during the intervention, i.e., the difference is that it is the emotional state of the moment that influences and guides the implementation of the art therapy, and therefore its effectiveness is more affected by the subject’s mental health in the present moment. The temporal dynamics of art therapy efficacy reveal critical implementation parameters. Emotion-driven modalities (e.g., drawing/writing) demonstrate strict adherence requirements—regularity and continuity are essential for therapeutic maintenance. However, implementation limitations were evident: short intervention durations (≤5 weeks) coupled with extended follow-up intervals created therapeutic discontinuities, potentially professional supervision continuity and compromising protocol obedience. Dance, music and yoga on the other hand are objective therapies to influence the improvement of the patient’s mood, which is less influenced by the factor of time and hence the intervention is more effective as compared to the former. Similarly in several studies there was little improvement in the fatigue index (25, 35). may be attributed to multiple interrelated factors: procedural memory decay of core therapeutic elements compromises intervention fidelity while post-intervention environmental stressors and weakened therapeutic alliance dependent on sustained therapist-patient interaction collectively erode clinical benefits. These temporal and contextual challenges collectively contribute to the attenuation of long-term fatigue-related benefits, highlighting the need for structured maintenance strategies to preserve therapeutic effects beyond the intervention period.

This meta-analysis also has some limitations. One is that only English articles were searched and the literature covers a limited area. Second, the outcome indicators were analyzed using scales, which can easily be influenced by the subjective consciousness of the participants. It is suggested that some laboratory or clinical indicators can be added to make the results more objective, so as to prove that the quality of life and fatigue of the patients can be improved, in order to increase the objectivity of the results.

As breast cancer management increasingly prioritizes psycho-oncological care, this meta-analysis establishes a precision framework for optimizing art therapy interventions. Time-dependent efficacy patterns reveal interventions require ≥2-month duration to achieve clinically meaningful quality of life improvement (SMD = 0.45-0.21, *P*<0.00001), while fatigue benefits demonstrate temporal decay beyond 6-month follow-up (SMD from -0.34 to 0.14). These findings necessitate two-phase intervention designs: intensive protocols (<6 months) for fatigue management and maintenance protocols for sustained quality of life. Population heterogeneity analysis identifies critical moderators: gender-specific neuroendocrine responses (exclusively female cohort) and age-stratified social cognition patterns (20-75 year range) may explain differential therapeutic outcomes, underscoring the need for personalized modality selection. Future trials should implement longitudinal designs with adaptive dosing strategies, incorporating gender-specific protocols and age-stratified subgroup analyses to maximize therapeutic precision.

## 5 Conclusion

Our research indicates that art therapy helps alleviate fatigue and enhance the quality of life for individuals with breast cancer. The quality of life is improved by long-term art therapy. Our research might provide patients with breast cancer an unusual approach to non-exercise rehabilitation. However, fatigue is more susceptible to the effects of time than quality of life; in a nutshell, fatigue has much improved in the short term, but it is not as noticeable in the long run. To enhance patients’ long-term fatigue, additional intervention programs must be created in the future.

## Data Availability

The original contributions presented in the study are included in the article/supplementary material. Further inquiries can be directed to the corresponding author/s.
